# Identification of “BRAF-Positive” Cases Based on Whole-Slide Image Analysis

**DOI:** 10.1155/2017/3926498

**Published:** 2017-04-24

**Authors:** Vlad Popovici, Aleš Křenek, Eva Budinská

**Affiliations:** ^1^Institute of Biostatistics and Analyses, Faculty of Medicine and Research Centre for Toxic Compounds in the Environment, Faculty of Science, Masarykova Univerzita, Kamenice 5, 625 00 Brno, Czech Republic; ^2^Institute of Computer Science, Masarykova Univerzita, Šumavská 15, 602 00 Brno, Czech Republic; ^3^Research Centre for Toxic Compounds in the Environment, Faculty of Science, Masarykova Univerzita, Kamenice 5, 625 00 Brno, Czech Republic

## Abstract

A key requirement for precision medicine is the accurate identification of patients that would respond to a specific treatment or those that represent a high-risk group, and a plethora of molecular biomarkers have been proposed for this purpose during the last decade. Their application in clinical settings, however, is not always straightforward due to relatively high costs of some tests, limited availability of the biological material and time, and procedural constraints. Hence, there is an increasing interest in constructing tissue-based surrogate biomarkers that could be applied with minimal overhead directly to histopathology images and which could be used for guiding the selection of eventual further molecular tests. In the context of colorectal cancer, we present a method for constructing a surrogate biomarker that is able to predict with high accuracy whether a sample belongs to the “BRAF-positive” group, a high-risk group comprising V600E BRAF mutants and BRAF-mutant-like tumors. Our model is trained to mimic the predictions of a 64-gene signature, the current definition of BRAF-positive group, thus effectively identifying histopathology image features that can be linked to a molecular score. Since the only required input is the routine histopathology image, the model can easily be integrated in the diagnostic workflow.

## 1. Introduction

The pathologic assessment of the tumor specimen provides the essential information for patient management, outcome estimation, and treatment decision. In the case of colorectal cancer (CRC), the main parameters of the pathologic assessment include the TNM stage, histologic grade, tumor type, vascular infiltration, and status of the resection margins [1]. Aside from these classical parameters, the discovery of molecular drivers and markers for resistance led to refined prognostic and predictive models [2]. For example, it has been shown that* KRAS*-mutated tumors are resistant to anti-*EGFR* treatment [3, 4]. In parallel several molecular taxonomies partially explaining intertumoral heterogeneity have been proposed for CRC [5–7]. Of interest for the current study is the identification of a high-risk group of CRC patients consisting of* V600E BRAF* mutants and a sizeable* BRAF*-wild type subset of tumors which display a similar pattern of gene activation, the so-called* BRAF*-mutant-like tumors [8]. This group is collectively called* BRAF*-positive, as the defining 64-gene signature has positive values for these cases [8]. These are only a few of the plethora of gene expression signatures proposed for CRC (in other types of cancer, the situation being similar) and they all have in common the requirement for profiling a rather large panel of genes and the limited usage in clinical practice. Among the reasons for their slow adoption are the associated costs for tests and limited availability of biological material. On the other hand, if one could robustly predict the outcome of some of these molecular tests directly from the data available for the pathologic assessment, significant speed-ups and cost cuts would be achieved. This is one of the main justifications of the present study, in which we propose an image analysis model for recognizing the “*BRAF*-positive” cases of CRC, that is, to predict the (dichotomized) outcome of the* BRAF* signature [8]. A second and broader in scope justification is the interest in identifying and understanding the connections between tumor architecture and gene activity as captured by transcriptomics.

Such connections between phenotypical appearance of the tumor and gene activity have been established before. For example, in the case of breast cancer the lobular phenotype is associated with deletions in the* CDH1* gene (encoding E-cadherin) [9] and the mesenchymal/metaplastic features are predictive in the case of* AR*-positive triple negative breast cancers [10]. In the case of colorectal cancer (CRC) the association of mucinous/serrated carcinomas with* BRAF* mutations is well known and we have shown that such association can be extended to the group of “*BRAF*-mutated-like” tumors, characterized by a specific genomic signature [8]. Similarly, connections between nuclear morphometry and molecular data have been identified in glioblastoma [11] and exploited in a multimodal prognostic signature in breast cancer [12]. When deriving molecular subtypes for colorectal cancer, we have also identified tumor architecture patterns preferentially enriched in those subtypes [5]. These observations all support the idea that genomic and phenotypic traits can be put in correspondence and, by consequence, that some phenotypic features could potentially be used as proxies for genomic markers.

In the present work, we propose an approach at building a histology image-based classifier able to predict the “*BRAF*-positive” status, as defined by the genomic signature. The gene expression data for the signature is supposed to be obtained from the same (or adjacent) tumor section as the histopathology whole-slide image. The key point of our approach resides in a convenient summarization of the imaging data into a code vector used for building the classification model. Apart from our own earlier results [13], there were no other studies to guide our selection of image features useful for this task. Hence, we took a data-driven approach in which the implicit hypothesis was that local tumor appearance contained enough information to build a predictor for the genomic “*BRAF*-positive” status. Thus, our approach was prior-free, in the sense that we did not restrict ourselves to a set of predefined (by an expert pathologist) measurements, with the potential drawback of limiting interpretability of the results.

Having a tissue-based surrogate biomarker for a genomic test allows an immediate integration in the routine diagnostic workflow and may provide the pathologist with hints for further genomic testing. This integration is supported by the increased adoption of digital pathology solutions. Additionally, such models can be applied to pathology image archives for the selection of cases for retrospective studies.

## 2. Materials and Methods

### 2.1. Data

The data collection used consisted of *n* = 291 samples for which both histopathology whole-slide images and clinical data (including* BRAF* and* KRAS* mutation status) were available, along with gene expression necessary for computing the* BRAF *score [8]. These samples were a subset of the data collected in the PETACC-3 clinical trial [14] and were selected based on the image quality and availability of the mutation information. A summary of the data is presented in Table 1 detailing the following clinical and molecular parameters, in this order: tumor stage; microsatellite stability status (high microsatellite instability (MSI-M) versus low microsatellite instability (MSI-L) or microsatellite stable (MSS)); mutation status of* BRAF* (V600E mutation) and* KRAS* (in codons 12 and 13) oncogenes; BRAF score (from the genomic signature) and the mucinous histology status of the tumor.

For each sample, a whole-slide image of haematoxylin-eosin (H&E) stained tumor sections was acquired at 20x magnification, using Hamamatsu NanoZoomer C9600 scanner. The resulting images were compressed by the image acquisition software using JPEG standard (at 80% quality) and stored in the proprietary NDPI format. The resolution of the images was 455 nm/pixel (equivalent to 55824 DPI) for a typical size of 100,000 × 50,000 pixels (varying with the size of the tissue section). The images were exported in standard TIFF format using OpenSlide software library [15].

### 2.2. Image Preprocessing

The whole-slide images were downscaled to an equivalent 5x magnification and only tumoral regions were retained from each sample (manually cut following the pathologist's annotations), the pixels outside the tumors being set to zero. To obtain the intensity signal corresponding to the haematoxylin and eosin dyes, the color deconvolution method from [16] was used, resulting in two single channel (intensity) images (H- and E-images).

### 2.3. Feature Extraction and Image Summarization

Our main assumption for image data modeling was that local appearance of the tissue section (local texture) contains enough information to yield discriminative features. However, the representation of an image in terms of a set of local descriptors still does not allow a direct comparison of two images (required for building a classifier); hence further summarization and standardization of the representation are needed. A suitable framework is represented by the image-retrieval applications based on Bag-of-Visual-Words methods [17]. In this framework, the local descriptors are used to construct a codebook for image representation (the information in the image is highly compressed) and the image is recoded in terms of frequencies of elements (visual codewords) from the codebook. We adapted this general approach to the problem at hand, as follows.

We decided to use a two-level approach to image representation with the first level (L1) being generic for all images and the second one (L2) specific to each class. The main reason behind this approach was that the first coding level was designed to capture the appearance of small structures (several cells, patches of stroma, parts of the colon crypts, etc.), while the second level was intended to capture larger arrangements of basic structures, which might be specific to each class. Additionally, since the classification problem was highly imbalanced, such separation would allow structures of both classes to be equally represented. Such multilevel approach has been already used in natural scene categorization [18]; however in our method we used the class label in generating the second level representation.

The first level (L1) of coding considered local patches of size 32 × 32 pixels as the basic processing unit. For such patches, we used the Gabor descriptors computed on both H- and E-images for each sample. These descriptors were based on the real component of the Gabor filter [19]:

(1) Gx,y;ν,θ,σ=exp⁡−x2+y22σ2×exp⁡2πνjxcos⁡θ+ysin⁡θ,

where $j=\sqrt{-1}$ and *ν* was the frequency, *θ* the orientation, and *σ* the bandwidth of the Gaussian kernel, respectively. The parameters were fixed throughout all experiments: $\sigma \in \left\{1,2\sqrt{2}\right\},\;\theta \in \left\{k\left(\pi /4\right)|k=0,\ldots ,3\right\}$, and *ν* ∈ {3/4, 3/8, 3/16}. In total, there were 24 Gabor filters that led to a 48-valued descriptor vector for each H- and E-image, with the first 24 values representing the mean response and the last 24 values representing the variance of the filter responses, over the considered 32 × 32 pixels' patch. Thus, to each local patch from the original images corresponded 96-value descriptor vectors obtained by concatenating the Gabor descriptors of the H- and E-images.

From each image in the training set (which will be generated within the cross-validation loop, see Classifier Design), 1,000 random patch descriptors were selected for building the L1 codebook using the standard* k*-means clustering, with *K*_1_ = 128 clusters. Then, all the patches were assigned a code 1,…, *K*_1_ based on the closest cluster (codeword) from the L1 codebook.

The second level of coding (L2) considered neighborhoods of 15 × 15 L1 patches (i.e., 480 × 480 pixels). For each such neighborhood, the descriptor computed was the vector of frequencies of the L1 codes (a vector with *K*_1_ values). Similarly to L1 coding, a new codebook was constructed by clustering L2 descriptors (500 random L2 descriptors selected from each image) with *K*_2_ = 128 clusters. Two such codebooks were constructed, one of each class (*BRAF*-positive and* BRAF*-negative), and then both used for coding each image, leading to a representation with codes 1,…, 2*K*_2_.

The process described above led to a recoding of each image in terms of a histogram with 2*K*_2_ bins, each corresponding to an L2 code. We note that, in all the steps for image coding, the patches containing more than 50% of background pixels were excluded.

### 2.4. Classifier Design

After the image recoding step, to each image corresponded a 2*K*_2_-value vector which constituted the input data for the classifier design. The classifier design included the following main steps:

1. Classifier feature selection: features (elements of the input vectors) were ordered based on recursive feature elimination (RFE) method [20] and subsets of features of sizes *f* = 30, 50,…, 130 (approximately half of total number of features) were considered for Step  (2).
2. For each subset of features, a Support Vector Machine (SVM) [21] with Radial Basis Function (RBF) kernel was trained and its metaparameters were optimized in an inner cross-validation loop. Its performance was estimated by cross-validation and the estimated area under the ROC curve (AUC) recorded.
3. The number of features yielding the maximum AUC was deemed optimal and the final SVM was trained on that number of features.

To estimate the performance of the system, the image recoding procedure followed by Steps  (1)–(3) above was embedded into an external 10-fold stratified cross-validation loop, thus ensuring an unbiased estimation. The vector of predicted labels within this outer cross-validation was taken to represent typical predictions of the model and used in statistical analyses to avoid overly optimistic conclusions that would have been obtained from the predictions made by the model trained on the full data set.

### 2.5. Statistical Analyses

The main performance parameter for the classifier was AUC, but sensitivity and specificity were equally measured. For sensitivity and specificity 95% confidence intervals were computed using Agresti-Coull approximation [22] while for AUC they were obtained by bootstrap [23]. To test the association between individual image features and the class label, univariable logistic regression models were fit and the sign of the resulting coefficient was used to determine the sense of the association. To test for the association between clinical variables and classifier predictions we used *χ*^2^-test on 2 × 2 contingency tables. Survival analysis was performed using survival package (version 2.39-4) from R statistical computing environment (version 3.3.1, http://www.r-project.org). The estimation of hazard ratios was obtained from Cox proportional hazards regression in the absence of any other covariates, while the comparison of survival experiences of different subgroups was assessed by log-rank test (Mantel-Haenszel test). Statistical significance level was chosen to be *p* = 0.05 and no adjustment for multiple hypotheses testing was performed.

## 3. Results and Discussion

### 3.1. Image-Based Predictor

The estimated performance of the classifier was AUC = 0.938, 95% CI = (0.903–0.972), with a default operating point yielding a sensitivity Se = 0.848, 95% CI = (0.733–0.920), and a specificity Sp = 0.926, 95% CI = (0.917–0.974), corresponding to an accuracy Acc = 0.931, 95% CI = (0.896–0.956). The optimal number of features varied throughout the cross-validation iterations between 70 and 110. In Table 2, the confusion matrix from the cross-validation predictions is shown.

**Figure 1:**
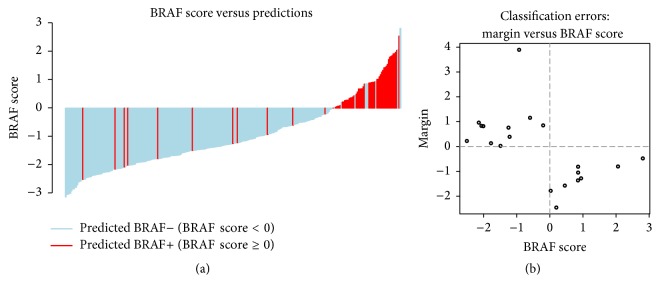
Analysis of the classifier's predictions. (a) Waterfall plot of the BRAF scores and the corresponding predictions (color-coded). (b) The relationship between the genomic score (*x*-axis) and the prediction margin (*y*-axis) for the misclassified samples.

The relationship between the image-based classifier predictions (from cross-validation) and the genomic score can be seen in Figure 1. The misclassified samples are covering the whole range of genomic scores (Figure 1(a)). For the SVMs, the margin of a sample can be viewed as a confidence in the prediction; hence we were interested in studying the classification errors in the context of their corresponding margins. In Figure 1(b), the margins are shown as a function of genomic score. It appears that smaller margin corresponds to larger (in absolute value)* BRAF* scores indicating that the confidence in those (erroneous) predictions is rather low.

A different trade-off between sensitivity and specificity could be obtained by adapting the classifier's threshold: for example, an operating point yielding Se = 0.915, 95% CI = (0.812–0.967), and Sp = 0.776, 95% CI = (0.718–0.825), would favor the detection of* BRAF*-positives.

### 3.2. Relationship with Clinical Parameters

Further investigation of the classifier's errors showed that most of the false negatives were* KRAS* mutants (6 out of 9) while the majority of the false positives were double wild type (*BRAF* and* KRAS* wild type). We also note that the classifier labeled two cases (out of 16) of* BRAF* mutant tumors as “BRAF-negative”; however, one of them had also a negative genomic score. The predictions were also associated with the mucinous status of the tumors (*χ*^2^ test *p*  value = 0.0066), the microsatellite instability status (*χ*^2^ test *p*  value < 0.0001), and the grade (*χ*^2^ test *p*  value = 0.0006) as expected [8] but not with other clinical parameters including* KRAS* mutation status and tumor stage.

The* BRAF* genomic signature was shown to have a strong prognostic value for overall survival (OS) and survival after relapse (SAR) and limited value for relapse-free survival (RFS) [8]. In the subset of samples considered, the genomic signature maintained its prognostic value and the classifier predictions inherited, to some degree, this property: the predictions were prognostic for OS (*p* = 0.007, HR = 1.81, 95% CI = (1.17–2.81)) and SAR (*p* = 0.010, HR = 1.89, 95% CI = (1.16–3.10)) but not for RFS (*p* = 0.072, HR = 1.44, 95% CI = (0.97–2.13)).

### 3.3. The Predictive Image Features

**Figure 2:**
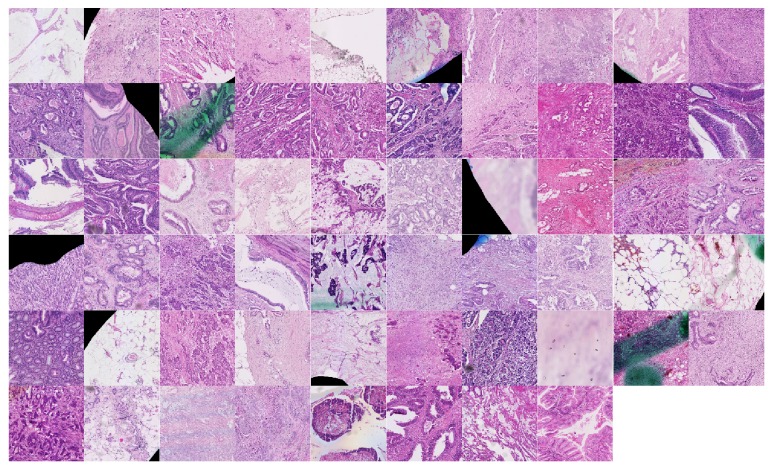
“Positive features”: image patterns associated with BRAF-positive class. Each feature is a 480 × 480 image patch and corresponds to an L2 codeword. Higher resolution image is available at DOI: 10.5281/zenodo.376999.

**Figure 3:**
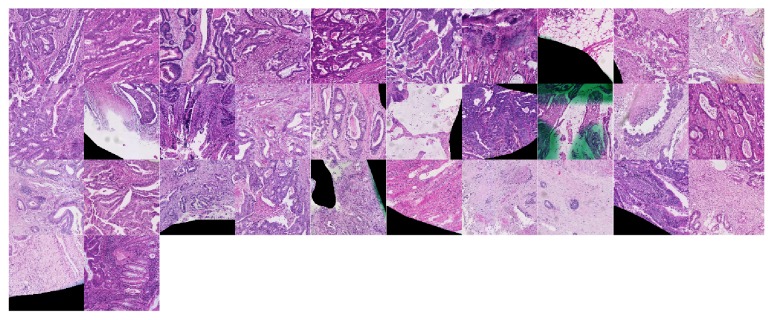
“Negative features”: image patterns associated with BRAF-negative class. Each feature is a 480 × 480 image patch and corresponds to an L2 codeword. Higher resolution image is available at DOI: 10.5281/zenodo.376999.

We investigated the structure of the final model generated using the complete data set, on which both image recoding and the classifier design steps were applied as described above. For this model, 90 features (corresponding to codewords from the L2 codebook) were selected as the optimal set and using the logistic regression coefficient (from single-variable models) they were divided into “positive features” (preferentially present in* BRAF*-positive cases, 58 features in total, see Figure 2) and “negative features” (preferentially present in* BRAF*-negative cases, 32 features in total, see Figure 3). We note that a number of features were dedicated to representing the border of the tumors and that some were partially affected by the markings present on the slides. It appears that the color deconvolution used in combination with Gabor descriptors made the representation robust to this type of noise. A second observation was that there were, roughly, twice as many image features representing the positive class compared to the negative one. This was to some degree not unexpected: indeed, in general, the* BRAF*-mutated and MSI-H CRC tumors show more intratumoral heterogeneity than the rest; however our results may suggest that this characteristic is common to a larger group of tumors.

**Figure 4:**
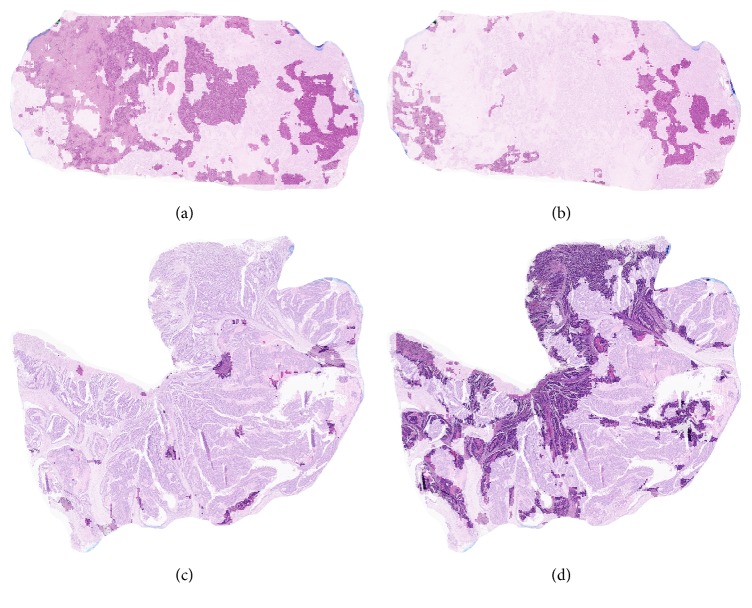
Spatial distribution of (positive and negative) features in two correctly classified images. The regions with low contrast were not involved in the classification process. (a-b) A* BRAF*-positive tumor: (a) positive image features; (b) negative image features. (c-d) A* BRAF*-negative tumor: (c) positive image features; (d) negative image features. Higher resolution images are available at DOI: 10.5281/zenodo.376999.

The exact contribution of each feature to the final decision is less obvious as their involvement in the classifier's prediction is through the RBF kernel and since the support vectors (actually a number of images from the training set) are defining the separation boundary between classes. However, a visualization of their spatial distribution in images may help in qualitatively understanding the model: in Figure 4 two examples of correctly classified tumors are shown. It appears that the features identified as “positive features” cover a relatively larger region in the* BRAF*-positive tumors than the “negative features.” The inverse relationship holds for the* BRAF*-negative tumors.

We also investigated whether the codebooks (for both levels of coding, L1 and L2) are biased towards one or a small group of images. We recall that the codebooks have been generated using an equal number of image patches randomly selected from the images. None of the clusters of the codebooks was dominated by a particular image, indicating that the codebooks capture general features.

## 4. Conclusions

We presented an image-based classifier that was able to predict with high accuracy the outcome of a genomic score. The input images were scans of H&E pathology slides making the system suitable for integration in the routine diagnostic procedures. Since the predictions of the classifier (as those of the corresponding genomic score) were not correlated with the TNM staging, they brought an independent indication of high-risk tumors (in the case of positive predictions). The system could also be applied for the retrospective selection of cases from tumor archives, reducing the volume of cases that an expert would need to evaluate.

Another important outcome is the observation that some gene expression based signatures may be translated into an image-based surrogate biomarker. Such tissue-based biomarkers may be used as a filtering step before the genomic tests.

## Figures and Tables

**Table 1:** Summary of main clinical parameters.

Parameter	*N*	Proportion (%)
Stage		
Stage II	55	18.9
Stage III	236	81.1
MSI		
MSI-H	12	4.1
MSI-L & MSS	279	95.6
V600E BRAF status		
Mutated	16	5.5
Wild type	275	94.5
KRAS (codons 12 and 13) status		
Mutated	113	38.8
Wild type	178	61.2
BRAF score		
Positive	59	20.3
Negative	232	79.7
Mucinous		
Yes	33	11.3
No	258	88.7

**Table 2:** Confusion matrix for classifier predictions. The ground truth is given by the genomic signature.

	Predicted BRAF-negative	Predicted BRAF-positive
Genomic BRAF-negative	221	11
Genomic BRAF-positive	9	50
